# Impact of dietary patterns on the survival outcomes of patients with cardiovascular disease

**DOI:** 10.3389/fnut.2025.1535174

**Published:** 2025-07-30

**Authors:** Jinyu Sun, Xingyu Jiang, Zheng Li, Yang Shen

**Affiliations:** ^1^Department of Cardiology, Suzhou Ninth People’s Hospital, Suzhou Ninth Hospital Affiliated to Soochow University, Suzhou, China; ^2^Gusu School, Nanjing Medical University, Suzhou, China; ^3^The First Affiliated Hospital of Nanjing Medical University, Nanjing, Jiangsu, China

**Keywords:** dietary patterns, cardiovascular disease, survival outcomes, weighted Cox regression, predictive ability

## Abstract

**Background:**

This study examines the association between dietary patterns and survival outcomes in patients with cardiovascular disease (CVD).

**Methods:**

A total of 9,101 adults with CVD from the 2005–2018 National Health and Nutrition Examination Survey were included. Dietary patterns were evaluated using five indices: the Alternative Healthy Eating Index (AHEI), Dietary Approaches to Stop Hypertension (DASH), Dietary Inflammatory Index (DII), Healthy Eating Index-2020 (HEI-2020), and the Alternative Mediterranean Diet Score (aMED). Associations between dietary indices and all-cause mortality were assessed using Kaplan-Meier survival analysis, weighted Cox regression models, and restricted cubic spline analyses. Predictive performance was evaluated using time-dependent receiver operating characteristic (Time-ROC) curves.

**Results:**

After a median follow-up of 7 years, 1,225 deaths were recorded. Survivors had higher AHEI, DASH scores, and lower DII scores. Kaplan-Meier analysis suggested better survival outcomes associated with higher adherence to healthier dietary patterns (AHEI, DASH, HEI-2020, aMED) and lower adherence to pro-inflammatory diets (DII). Weighted Cox regression revealed significant associations between higher scores on AHEI, DASH, HEI-2020, and aMED and reduced mortality risk (highest vs. lowest tertile HRs: 0.59, 0.73, 0.65, and 0.75, respectively; all *P* < 0.05). Conversely, higher DII scores were associated with increased mortality risk, with the highest tertile showing significantly elevated risk compared to the lowest tertile (HR = 1.58, 95% CI: 1.21–2.06; *P* < 0.001). Restricted cubic spline analyses identified a significant non-linear relationship between AHEI scores and mortality (P _*for*_ _*non–linearity*_ = 0.036), while other indices exhibited linear associations. Time-ROC analysis indicated that dietary indices maintain relatively consistent predictive effectiveness for mortality risk over time.

**Conclusion:**

Improved healthy dietary patterns could potentially reduce mortality risk in CVD patients, underscoring the need for dietary quality enhancement in managing CVD.

## 1 Introduction

Cardiovascular disease (CVD) is one of the major leading causes of morbidity and mortality worldwide, posing a substantial public health challenge (1). Among modifiable risk factors, dietary patterns play an important role in cardiovascular health by influencing key metabolic and inflammatory pathways (2, 3). To better understand these relationships, several dietary indices have been developed to assess overall diet quality and its impact on health outcomes. Prominent examples include the Dietary Inflammatory Index (DII), the Alternative Healthy Eating Index (AHEI), the Dietary Approaches to Stop Hypertension (DASH), the Healthy Eating Index-2020 (HEI-2020), and the alternative Mediterranean Diet Score (aMED) (4–7). Each of these indices captures distinct yet complementary aspects of diet composition and quality (5–8).

Dietary research in the context of CVD has evolved through landmark studies, shaping current knowledge of dietary recommendations (9, 10). For instance, a study spanning 80 countries highlighted the critical role diet plays in cardiovascular health, revealing population-specific differences in diet-related CVD outcomes (10). Subsequent research, including the DASH trial and studies on the aMED, further established the association between specific dietary patterns and reduced CVD risk (11–16). Collectively, these findings have provided the foundation for dietary guidelines focused on CVD prevention, highlighting the value of adopting healthier eating patterns.

Although previous studies have established the association between diet and CVD risk, most have focused primarily on single dietary pattern or individual nutrients, rather than comprehensively evaluating multiple dietary indices. Moreover, limited research has explored the prognostic value of these dietary indices for mortality within large population-based cohorts. Accordingly, a systematic assessment of multiple dietary indices is essential to elucidate their relative contributions to CVD outcomes.

This study investigates the associations between five widely used dietary indices and mortality risk among patients with CVD, using data from a large, nationally representative cohort. Specifically, we examine whether adherence to healthier dietary patterns (AHEI, DASH, HEI-2020, aMED) is associated with improved prognosis, and conversely, whether pro-inflammatory diets (DII) are associated with poorer outcomes. We hypothesize that higher adherence to AHEI, DASH, HEI-2020, and aMED patterns correlates with a reduced risk of mortality, while higher DII scores predicts an elevated mortality risk.

## 2 Materials and methods

### 2.1 Study population

**FIGURE 1:**
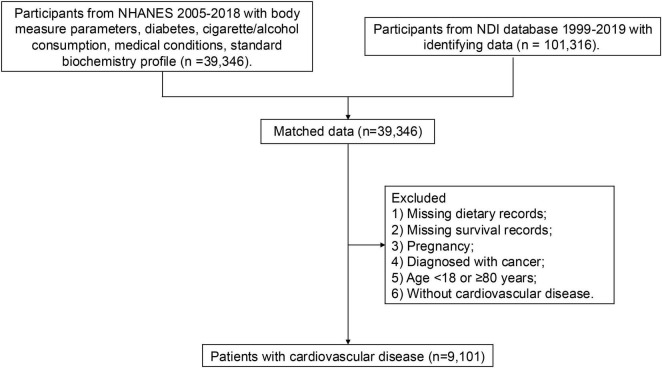
Flow chart of selection of eligible participants.

The National Health and Nutrition Examination Survey (NHANES) is an ongoing cross-sectional study conducted by the National Center for Health Statistics. Since 1999, the continuous NHANES has been conducted in two-year cycles, employing a complex stratified multistage clustered sampling design to ensure national representativeness. This study included participants from seven NHANES cycles: 2005–2006, 2007–2008, 2009–2010, 2011–2012, 2013–2014, 2015–2016, and 2017–2018. Participants meeting any of the following criteria were excluded: (1) missing dietary records, (2) missing survival records, (3) pregnancy, (4) diagnosed with cancer, (5) age younger than 18 or older than or equal to 80 years, and (6) without CVD (Figure 1). Finally, a total of 9,109 were included in the following analysis. All participants provided informed consent. NHANES protocols were approved by the National Center for Health Statistics Research Ethics Review Board.

### 2.2 Assessment of diet quality

NHANES survey collects dietary intake during the last 24 h before interview, and “Dietaryindex” package was applied to calculate the five diet indices (17). The DII is a scoring algorithm developed based an extensive literature review spanning 1950–2010. DII assesses the inflammatory potential of diets by evaluating the relationship between 45 food parameters and six inflammatory biomarkers, including interleukin (IL)-1, IL-4, IL-6, IL-10, tumor necrosis factor-alpha, and C-reactive protein. Each dietary parameter is assigned a score reflecting its pro-inflammatory (+ 1), anti-inflammatory (−1), or neutral (0) influence on these biomarkers. The DII score ranges from + 7.98 (most pro-inflammatory) to −8.87 (most anti-inflammatory), with high scores indicating greater inflammatory potential (18).

The AHEI was developed based on clinical and epidemiological evidence linking specific dietary components to chronic disease risk. This scoring system comprises 11 dietary components, each rated from 0 (least healthy) to 10 (most healthy), producing a total score ranging between 0–110. Components promoting higher scores include greater intakes of vegetables, fruits, whole grains, nuts, legumes, long-chain omega-3 fatty acids, and polyunsaturated fatty acids, alongside lower consumption of sugar-sweetened beverages, fruit juices, red and processed meats, trans fats, sodium, and alcohol. Higher AHEI scores reflect greater adherence to dietary patterns associated with reduced chronic disease risk.

The DASH score comprises eight key dietary components, each categorized into quintiles and assigned a score from 1 (lowest adherence) to 5 (highest adherence). This system favors higher intakes of fruits, vegetables, nuts, legumes, low-fat dairy products, and whole grains, while encouraging lower consumption of sodium, sugar-sweetened beverages, and red/processed meats. The total DASH score ranges from 8 to 40, with higher scores indicating greater adherence to the DASH dietary guidelines.

The HEI-2020 aligns with the 2020–2025 Dietary Guidelines for Americans, which consists of nine adequacy components (e.g., fruits, vegetables, grains, dairy, proteins, and fatty acids) and four moderation components (refined grains, sodium, saturated fats, and added sugars) (19). Higher consumption leads to higher scores for adequacy components, whereas lower consumption results in higher scores for moderation components, thus achieving a balance between dietary adequacy and moderation. The HEI-2020 score ranges from 0 to 100, with higher scores indicating better adherence to dietary recommendations.

The aMED score assesses adherence to the Mediterranean diet by evaluating nine dietary components intakes, including vegetables, fruits, whole grains, nuts, legumes, fish, red meats, alcohol, and fat quality (ratio of monounsaturated to saturated fatty acids). Participants who consumed above-median amounts of these components (except for red and processed meats) receive 1 point per component. Additionally, one point is awarded for below-median consumption of red and processed meats and for moderate alcohol intake (5–15 g/day for women, 10–25 g/day for men). The total aMED score ranges from 0 to 9, with higher scores reflecting greater adherence to the Mediterranean diet.

### 2.3 The definition of cardiovascular disease

All participants were asked about their medical history related to cardiovascular conditions, including angina pectoris, coronary heart disease, heart attack, heart failure, hypertension, and stroke. Individuals reporting a history of any of these conditions were classified as having CVD.

### 2.4 Assessment of mortality

NHANES data were linked to the publicly accessible National Death Index mortality file, which includes vital status and cause-of-death information for NHANES participants up to December 31, 2019. The National Center for Health Statistics determined all-cause mortality using a probabilistic matching algorithm to link NHANES participants with corresponding death certificates. Participants were followed death or until the end of the study period.

### 2.5 Covariates

Covariates were applied following previous studies exploring diet-CVD relationships (20–23). The following covariates were included: age (years), race/ethnicity (Hispanic, non-Hispanic White, non-Hispanic Black, other Hispanic, or others), Gender (male, or female), family income-to-poverty ratio (< 1.33, 1.33∼3.5, or ≥ 3.5), body mass index (kg/m^2^), waist circumference (cm), triglycerides (mg/dL), Cholesterol (mmol/L), estimated glomerular filtration rate (eGFR, mL/min/1.73m^2^), diabetes (yes, or no), smoking (yes, or no), and drinking (yes, or no). Diabetes were defined as fasting plasma glucose ≥ 126 mg/dL, hemoglobin A1c ≥ 6.5%, or self-reported diagnosis of diabetes.

### 2.6 Statistical analysis

Missing data were addressed using multiple imputation to minimize potential bias (24). Since the NHANES survey applied a multi-stage stratified random sampling approach, weighted statistical methods were applied appropriately in the following analysis. Continuous variables were presented as weighted medians with interquartile ranges, and categorical variables were expressed as weighted percentages. Participant characteristics were compared based on survival status using Kruskal-Wallis test for continuous variables and the chi-square test for categorical variables.

Kaplan-Meier curves were employed to illustrate survival rates among patients with CVD according to dietary indices categorized into tertiles, and survival probabilities were compared across these tertiles. Weighted Cox regression was applied to evaluate the association between each dietary index and all-cause mortality, using the lowest tertile as the reference category. Various known potential confounders of CVD prognosis was adjusted for, including age, gender, race/ethnicity, family income-to-poverty ratio, waist circumference, body mass index, triglycerides, estimated glomerular filtration rate, diabetes, smoking, and drinking. The results are presented as hazard ratios (HRs) with 95% confidence intervals (CIs).

Additionally, stratified analysis were performed by sex (male vs. female), diabetes status (present vs. absent), and BMI (< 25, 25–30, or ≥ 30 kg/m^2^). Sensitivity analyses were performed by excluding patients with severe renal impairment (eGFR < 30 mL/min/1.73m^2^). It should be noted that due to the observational and cross-sectional design, Cox regression analysis cannot establish the causal relationships between dietary patterns and CVD survival outcomes.

Moreover, restricted cubic splines were employed to visually represent the associations between dietary indices and mortality risk, using the median value of each dietary index as the reference point. To evaluate the predictive accuracy of the dietary indices across different models, time-dependent receiver operating characteristic (Time-ROC) curve analyses were conducted. *P*-value < 0.05 was statistically significant. All analyses were conducted using R software (R Foundation for Statistical Computing, Vienna, Austria).

## 3 Results

### 3.1 Participant characteristics

After a median follow-up of 7 years, a total of 1,225 deaths were documented among participants. Significant differences were observed between survivors and non-survivors in dietary index scores (Table 1). Specifically, non-survivors exhibited lower median scores for the AHEI (35.1 *vs.* 37.1, *P* < 0.001) and HEI-2020 (48.8 *vs.* 50.0, *P* = 0.048), and higher median scores for DII (2.1 *vs.* 1.6, *P* < 0.001), where as no significant differences were observed in DASH and aMED scores. Additionally, non-survivors showed decreased family income-to-poverty ratios, and a higher prevalence of diabetes, hypertension, smoking, alcohol consumption, and elevated triglyceride levels.

**TABLE 1:** The weighted baseline characteristics of the participants.

	Overall (*n* = 9,101)	Alive (*n* = 7,876)	Deceased (*n* = 1,225)	P
AHEI	36.8 (29.7, 44.9)	37.1 (29.7, 45.4)	35.1 (29.0, 42.0)	<0.001
DASH	26.5 (24.5, 28.5)	26.5 (24.5, 28.5)	26.0 (24.5, 28.0)	0.19
DII	1.7 (0.4, 2.7)	1.6 (0.3, 2.7)	2.1 (0.9, 3.0)	<0.001
HEI-2020	49.9 (42.5, 58.8)	50.0 (42.5, 59.0)	48.8 (42.4, 57.1)	0.048
aMED	5.5 (5.0, 6.5)	5.5 (5.0, 6.5)	5.5 (5.0, 6.5)	0.149
Age (years)	56.0 (46.0, 65.0)	55.0 (45.0, 64.0)	65.0 (57.0, 73.0)	<0.001
Race (%)				<0.001
Mexican American	6.4	6.6	4.8	
Non-Hispanic Black	15.6	15.6	16.3	
Non-Hispanic White	66.2	65.6	72.0	
Other Hispanic	4.6	4.8	3.0	
Other races	7.2	7.4	3.9	
Gender (male, %)	50.0	49.2	53.2	0.05
PIR level (%)				<0.001
<1.33	22.1	20.8	32.4	
1.33∼3.5	33.3	32.4	40.4	
≥3.5	44.6	46.8	27.2	
Body mass index (kg/m^2^)	30.8 (26.6, 35.9)	30.8 (26.7, 35.8)	30.8 (26.1, 39.8)	0.46
Waist circumference (cm)	106.2 (95.5, 118.2)	105.7 (95.5, 117.3)	111.4 (96.1, 127.0)	0.02
Triglycerides (mg/dl)	147.0 (97.0, 228.0)	146.0 (97.0, 226.0)	162.0 (98.0, 261.0)	0.21
Cholesterol (mmol/L)	5.0 (4.3, 5.8)	5.0 (4.3, 5.8)	4.9 (4.1, 5.9)	0.003
eGFR (mL/min/1.73m^2^)	109.4 (83.2, 145.6)	111.0 (85.7, 146.4)	90.0 (65.2, 136.7)	<0.001
Diabetes (Yes, %)	26.6	25.0	41.2	<0.001
Hypertension (Yes, %)	94.5	95.2	89.2	<0.001
Smoking (Yes, %)	50.5	48.6	66.0	<0.001
Drinking (Yes, %)	11.3	10.7	16.4	<0.001

AHEI, alternative healthy eating index 2010; DASH, dietary approaches to stop hypertension; DII, dietary inflammatory index; HEI-2020, healthy eating index-2020; aMED, alternate mediterranean diet score; PIR, family income-to-poverty ratio; eGFR, estimated glomerular filtration rate.

### 3.2 Dietary indices and mortality

**FIGURE 2:**
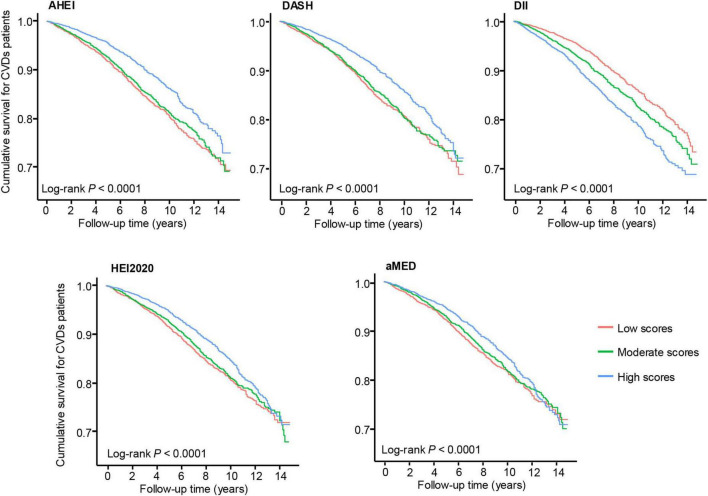
Kaplan-Meier survival curves for patients with cardiovascular disease according to dietary indices categorized into tertiles. Log-rank *P*-values for all indices are < 0.0001. The red, green, and blue lines represent low, moderate, and high dietary scores, respectively.

Kaplan-Meier survival curves were constructed to illustrate the associations between dietary index scores and survival outcomes among patients with CVD, stratified by dietary index tertiles (Figure 2). Higher adherence to healthier dietary patterns, including AHEI, HEI-2020, DASH, and aMED, was associated with improved survival. Conversely, higher DII scores demonstrated an inverse association, with greater inflammatory dietary potential linked to worse survival outcomes.

Subsequently, weighted Cox regression analyses were performed to quantify the associations between dietary indices and all-cause mortality, adjusting for potential confounders (Table 2). In the adjusted models, higher scores on dietary indices reflecting healthier dietary patterns were significantly associated with reduced mortality risk: AHEI (HR per unit increase = 0.98, 95% CI: 0.97–0.99), DASH (HR = 0.95, 95% CI: 0.92–0.98), and HEI-2020 (HR = 0.98, 95% CI: 0.97–0.99). Categorical analysis demonstrated that participants in the highest tertile (T3) of the AHEI (HR = 0.59, 95% CI: 0.47–0.75), DASH (HR = 0.73, 95% CI: 0.56–0.94), HEI-2020 (HR = 0.65, 95% CI: 0.50–0.85), and aMED (HR = 0.75, 95% CI: 0.60–0.95) exhibited significantly lower mortality risks compared to those in the lowest tertile (T1). Conversely, higher DII scores, indicative of more pro-inflammatory dietary patterns, were associated with increased mortality risk both continuously (HR = 1.15 per unit increase, 95% CI: 1.07–1.24) and categorically, with the highest tertile showing significantly elevated risk compared to the lowest tertile (HR = 1.58, 95% CI: 1.21–2.06).

**TABLE 2:** The association between diet indices and all-cause mortality based on weighted Cox regression.

	Non-adjusted model		Adjusted model	
	HR (95% CI)	*P*	HR (95% CI)	*P*
**AHEI**				
Continuous	0.98 (0.98, 0.99)	<0.001	0.98 (0.97, 0.99)	<0.001
**Categories**				
T1	Reference		Reference	
T2	0.96 (0.81, 1.15)	0.685	0.88 (0.72, 1.07)	0.186
T3	0.66 (0.53, 0.81)	<0.001	0.59 (0.47, 0.75)	<0.001
**DASH**				
Continuous	0.96 (0.93, 0.99)	0.015	0.95 (0.92, 0.98)	<0.001
**Categories**				
T1	Reference		Reference	
T2	0.93 (0.75, 1.16)	0.538	0.89 (0.69, 1.15)	0.365
T3	0.80 (0.63, 1.02)	0.068	0.73 (0.56, 0.94)	0.013
**DII**				
Continuous	1.18 (1.11, 1.25)	<0.001	1.15 (1.07, 1.24)	<0.001
**Categories**				
T1	Reference		Reference	
T2	1.33 (1.07, 1.65)	0.01	1.24 (0.98, 1.57)	0.073
T3	1.75 (1.41, 2.17)	<0.001	1.58 (1.21, 2.06)	<0.001
**HEI-2020**				
Continuous	0.99 (0.99, 1.00)	0.019	0.98 (0.97, 0.99)	<0.001
**Categories**				
T1	Reference		Reference	
T2	0.96 (0.80, 1.16)	0.68	0.85 (0.67, 1.08)	0.175
T3	0.82 (0.66, 1.01)	0.06	0.65 (0.50, 0.85)	0.002
**aMED**				
Continuous	0.90 (0.83, 0.98)	0.017	0.86 (0.77, 0.95)	0.003
**Categories**				
T1	Reference		Reference	
T2	0.86 (0.69, 1.06)	0.147	0.83 (0.65, 1.07)	0.148
T3	0.84 (0.70, 1.01)	0.061	0.75 (0.60, 0.95)	0.016

Adjusted model: We adjusted for age, gender, race/ethnicity, family income-to-poverty ratio, waist circumference, body mass index, triglycerides, estimated glomerular filtration rate, diabetes, smoking, and drinking. AHEI, alternative healthy eating index 2010; DASH, dietary approaches to stop hypertension; DII, dietary inflammatory index; HEI-2020, healthy eating index-2020; aMED, alternate mediterranean diet score.

Moreover, stratified analysis were further performed by sex (male vs. female), diabetes status (present vs. absent), and BMI categories to assess subgroup effects (Supplementary Figure 1). Although the general trends remained consistent across most subgroups, no significant associations were observed between dietary indices and survival outcomes in participants with diabetes, suggesting that diabetes status may modify or attenuate the observed relationships. Additionally, sensitivity analyses excluding participants with severe renal impairment yielded consistent results (Supplementary Table 1).

**FIGURE 3:**
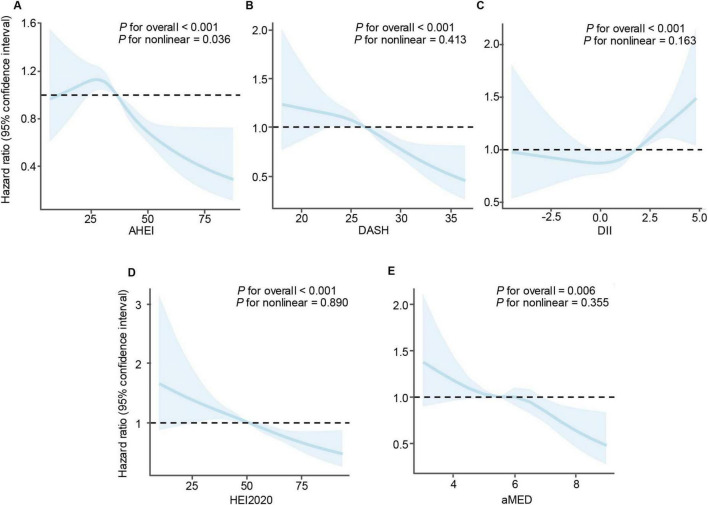
Restricted cubic spline analyses illustrating the association between dietary indices and mortality risk in patients with cardiovascular disease. (A) Alternative healthy eating index, (B) dietary approaches to stop hypertension, (C) dietary inflammatory index, (D) healthy eating index-2020, and (E) mediterranean diet score. A non-linear relationship was observed for AHEI, while linear associations were found for the other dietary indices.

Furthermore, restricted cubic spline were performed to evaluate potential non-linear associations between dietary indices and CVD mortality risk (Figure 3). The results indicated a significant non-linear relationship between the AHEI score and CVD mortality (P _*for*_ _*non–linearity*_ = 0.036), suggesting that the protective effect of higher AHEI scores does not increase uniformly. In contrast, no significant non-linear associations were observed for DASH (P _*for*_ _*non–linearity*_ = 0.413), HEI-2020 (P _*for*_ _*non–linearity*_ = 0.890), or aMED (P _*for*_ _*non–linearity*_ = 0.355) scores, indicating consistent relationships across their respective ranges. Similarly, the DII showed no significant non-linear relationship with mortality risk (P _*for*_ _*non–linearity*_ = 0.163).

### 3.3 Comparison of the predictive power of dietary indices

**FIGURE 4:**
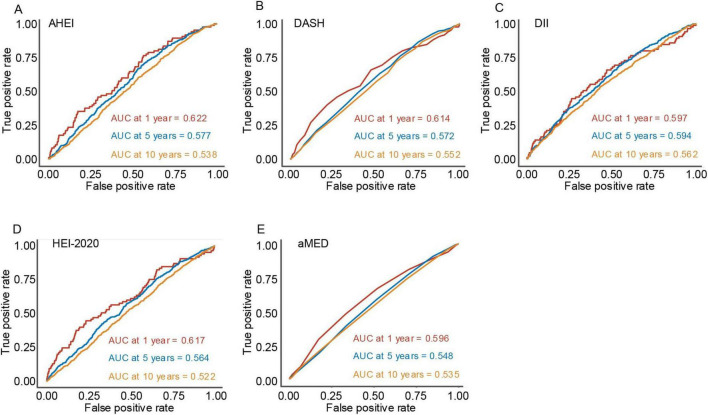
The plots depict the time-dependent area under the receiver operating characteristic curve at 1-year, 5-year, and 10-year follow-up periods for five dietary indices. (A) Alternative healthy eating index, (B) dietary approaches to stop hypertension, (C) dietary inflammatory index, (D) healthy eating index-2020, and (E) mediterranean diet score.

Time-ROC analyses were employed to assess the predictive accuracy of various dietary indices for overall survival, measured by the area under the time-dependent ROC curve (Time-AUC) (10). Figure 4 illustrates the predictive performance of these dietary indices at 2-, 5-, and 10-year intervals. Among the evaluated indices, all demonstrated modest predictive ability, with comparable TimeAUC values observed across the follow-up periods. These findings suggest that dietary indices maintain relatively consistent predictive effectiveness for mortality risk over time.

## 4 Discussion

By utilizing nationally representative NHANES data, our findings indicate complex association between diet quality and survival outcomes in patients with CVD, highlighting the protective role of healthier dietary patterns. Higher adherence to the AHEI, DASH, HEI-2020, and aMED diets correlated significantly with reduced risk of CVD mortality, aligning with existing evidence advocating the incorporation of healthy dietary patterns into public health interventions aimed at improving cardiovascular prognosis (25, 26). Specifically, dietary indices emphasizing higher consumption of fruits, vegetables, whole grains, nuts, legumes, and polyunsaturated fats, and lower intake of processed meats, sugary beverages, and sodium (such as DASH, HEI-2020, and aMED) have consistently demonstrated cardioprotective effects (27–29). Notably, we identified a non-linear relationship between the AHEI and mortality risk, suggesting a threshold effect beyond which incremental dietary improvements confer diminishing survival benefits. This non-linearity warrants consideration in future dietary guidelines and merits further investigation.

This study revealed a significant positive association between DII and mortality in patients with CVD, indicating that higher DII scores (more pro-inflammatory diets) corresponded with increased mortality risk. These findings align with previous research demonstrating similar associations between higher DII scores and adverse health outcomes. For example, the EPIC (European Prospective Investigation into Cancer and Nutrition) cohort study reported a positive association between higher DII scores and thyroid disease risk (30). Additionally, a previous analysis utilizing NHANES data found higher DII scores were linked to elevated risks of all-cause and CVD mortality among adults with hyperuricemia (31). Collectively, these findings reinforce the detrimental role of pro-inflammatory dietary patterns, further highlighting dietary inflammation as a modifiable risk factor that influences cardiovascular disease prognosis and mortality.

Our findings regarding the associations of dietary indices with CVD mortality could be partly explained by distinct underlying mechanisms. For example, dietary patterns such as DASH and AHEI may confer cardiovascular benefits by enhancing blood pressure regulation, improving lipid profiles, and reducing systemic inflammation, all of which have been previously linked to improved cardiovascular prognosis. In contrast, higher DII scores may reflect dietary components that promote chronic inflammation, a known factor in the progression and exacerbation of cardiovascular disease. Pro-inflammatory diets have been shown to elevate levels of circulating inflammatory biomarkers, potentially accelerating endothelial dysfunction, plaque formation, and subsequent cardiovascular events. Therefore, dietary indices reflecting inflammation, such as the DII, could serve as useful tools for identifying individuals at greater risk of adverse cardiovascular outcomes. Future research integrating inflammatory biomarkers and mechanistic studies may further elucidate these pathways, enabling the development of targeted nutritional strategies for CVD prevention and management.

Consistent with the pervious findings (13), our study confirmed that a higher HEI-2020 score is associated with lower CVD risk. However, we further identified a non-linear association for AHEI. This finding suggests that the benefits associated with improved dietary quality may not increase uniformly across the full range of dietary scores. Specifically, beyond a certain threshold, further increases in AHEI scores do not appear to yield additional substantial reductions in mortality risk. This non-linear relationship aligns with recent evidence indicating threshold effects, wherein health improvements plateau after reaching a certain level of dietary quality (32, 33). For instance, while traditional dietary guidelines typically suggest a proportional relationship between adherence to recommended dietary patterns and improved health outcomes, our results challenge this assumption, suggesting instead that optimal dietary quality, rather than maximal adherence, may be more critical in reducing disease risk and mortality (34). Similarly, previous studies have reported non-linear associations, suggesting that incremental dietary improvements beyond a certain threshold may confer limited additional benefits (32, 33). Therefore, public health guidelines and nutritional recommendations should shift toward emphasizing optimized, individualized dietary quality rather than universal adherence targets. Such an approach could enable the development of more tailored and potentially more effective public health nutrition strategies, particularly among populations already experiencing nutritional deficiencies.

Although this study systematically evaluates the association between multiple dietary indices and CVD prognosis, some limitations should be acknowledged. The cross-sectional design of the NHANES survey impedes the establishment of causal relationships. Additionally, dietary assessments relying on self-reported data are subject to recall and reporting biases. Although our analysis have adjusted for multiple confounders, the residual confounding from unmeasured variables (such as dietary supplement use, physical activity, and stress levels) cannot be excluded. Furthermore, dietary patterns are likely to evolve over time, yet the NHANES dataset captures dietary information only at a single point, limiting the evaluation of dietary changes. Following studies should adopt a longitudinal approach to monitor dietary pattern over time, which could provides deeper insights into their dynamic relationship with CVD outcomes. In particular, incorporating inflammatory biomarkers could further validate the predictive value of DII and elucidate its biological effect in the progression of CVD. Considering the global prevalence of CVD, it is crucial to investigate the impact of dietary modifications across various populations and contexts, which should facilate to develop specific dietary recommendations and effective public health strategies.

## 5 Conclusion

Our findings suggest that high scores on dietary indices such as AHEI, DASH, HEI-2020, and aMED are associated with reduced CVD mortality. In addition, we found a non-linear relationship between diet quality and CVD mortality, suggesting that there may be an optimal range of diet quality in actual clinical practice. These results emphasize the need for individualized nutritional guidance in clinical and public health contexts.

## Data Availability

The datasets presented in this study can be found in online repositories. The names of the repository/repositories and accession number(s) can be found in the article/Supplementary material.

## Supplementary material

Supplementary Figure 1 The association between diet indices and all-cause mortality based on weighted Cox regression stratified by sex (male vs. female), diabetes status (present vs. absent), and BMI (< 25, 25–30, or ≥ 30 kg/m^2^).
