# Supplementary material for: Impact of dietary patterns on the survival outcomes of patients with cardiovascular disease
Source: Front Nutr. 2025 Jul 30;12:1535174. doi: 10.3389/fnut.2025.1535174 (PMC12343251; doi:10.3389/fnut.2025.1535174)
Supplement: Supplementary file 1 [file Table_1.DOCX]

**Supplementary Table 1**. The association between diet indices and all-cause mortality in CVD patients with eGFR ≥ 30 mL/min/1.73m² based on weighted Cox regression.

|  | **Adjusted model** | |
| --- | --- | --- |
|  | HR (95% CI) | *P* |
| **AHEI** |  |  |
| Continuous | 0.98 (0.97, 0.99) | < 0.001 |
| Categories |  |  |
| T1 | *Reference* |  |
| T2 | 0.86 (0.70, 1.05) | 0.138 |
| T3 | 0.59 (0.46, 0.74) | <0.001 |
| **DASH** |  |  |
| Continuous | 0.95 (0.92, 0.98) | 0.002 |
| Categories |  |  |
| T1 | *Reference* |  |
| T2 | 0.87 (0.67, 1.14) | 0.324 |
| T3 | 0.73 (0.56, 0.95) | 0.018 |
| **DII** |  |  |
| Continuous | 1.15 (1.07, 1.24) | < 0.001 |
| Categories |  |  |
| T1 | *Reference* |  |
| T2 | 1.22 (0.96, 1.56) | 0.104 |
| T3 | 1.57 (1.20, 2.05) | 0.001 |
| **HEI-2020** |  |  |
| Continuous | 0.98 (0.97, 0.99) | < 0.001 |
| Categories |  |  |
| T1 | *Reference* |  |
| T2 | 0.83 (0.66, 1.06) | 0.129 |
| T3 | 0.65 (0.50, 0.85) | 0.002 |
| **aMED** |  |  |
| Continuous | 0.85 (0.77, 0.94) | 0.002 |
| Categories |  |  |
| T1 | *Reference* |  |
| T2 | 0.83 (0.64, 1.06) | 0.140 |
| T3 | 0.74 (0.59, 0.94) | 0.012 |

We adjusted for age, gender, race/ethnicity, family income-to-poverty ratio, waist circumference, body mass index, triglycerides, estimated glomerular filtration rate, diabetes, smoking, and drinking.

*Abbreviations: AHEI: Alternative Healthy Eating Index 2010; DASH: dietary approaches to stop hypertension; DII: dietary inflammatory index; HEI-2020: healthy eating index-2020; aMED: alternate mediterranean diet score.*
