# Supplementary figures and images for: Impact of dietary patterns on the survival outcomes of patients with cardiovascular disease
Source: Front Nutr. 2025 Jul 30;12:1535174. doi: 10.3389/fnut.2025.1535174 (PMC12343251; doi:10.3389/fnut.2025.1535174)

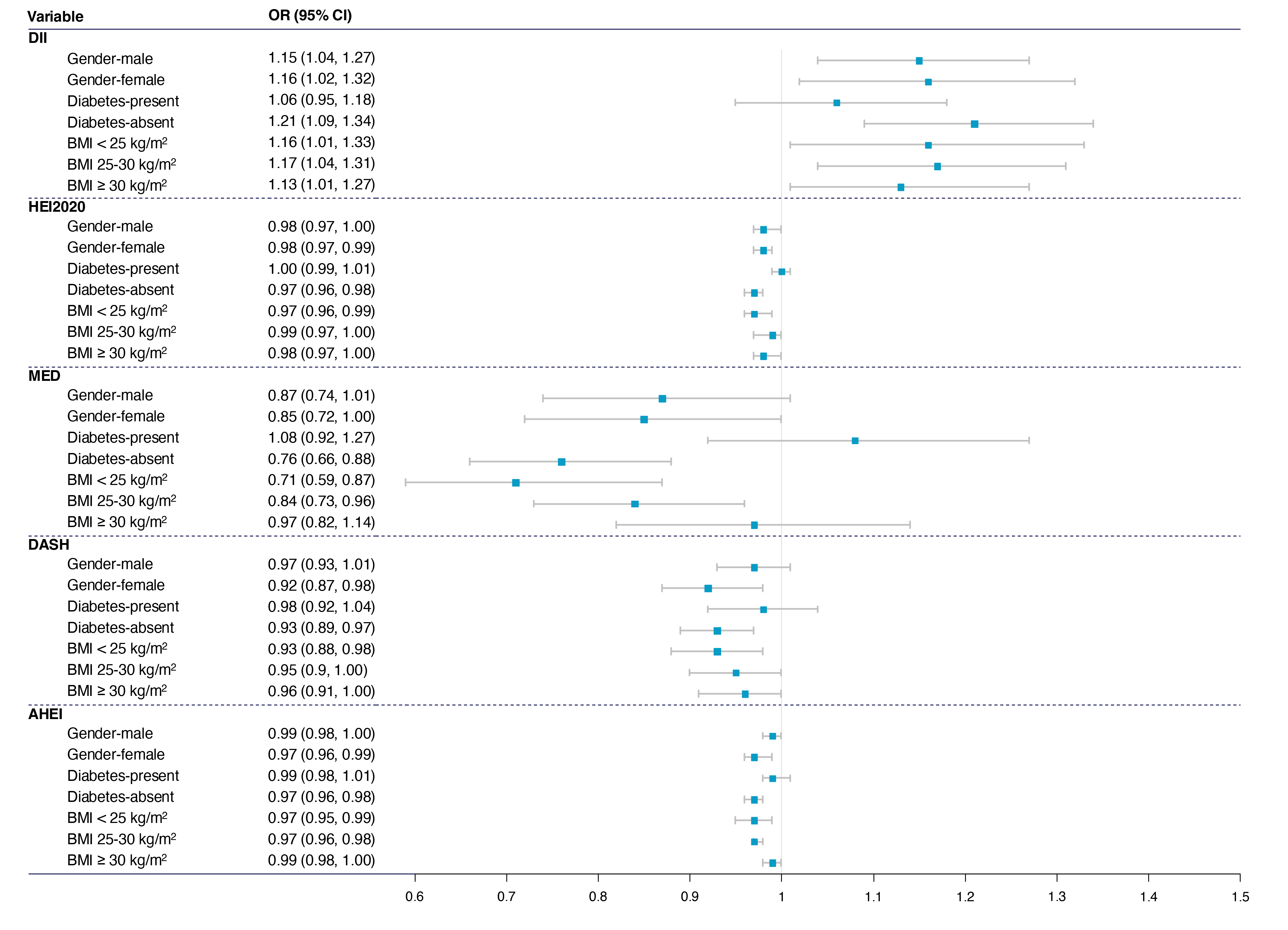

Supplement: Supplementary Figure 1 — The association between diet indices and all-cause mortality based on weighted Cox regression stratified by sex (male vs. female), diabetes status (present vs. absent), and BMI (< 25, 25–30, or ≥ 30 kg/m2). [file Image_1.tif]
